# *N*′-(1-Allyl-2-oxoindolin-3-yl­idene)benzohydrazide

**DOI:** 10.1107/S1600536810024918

**Published:** 2010-07-03

**Authors:** Abdulsalam Alsubari, Ahmed Moussaif, Hafid Zouihri, El Mokhtar Essassi, Seik Weng Ng

**Affiliations:** a Laboratoire de Chimie Organique Hétérocyclique, Pôle de Compétences Pharmacochimie, Université Mohammed V-Agdal, BP 1014 Avenue Ibn Batout, Rabat, Morocco; b CNRST Division UATRS, Angle Allal Fassi/FAR, BP 8027 Hay Riad, Rabat, Morocco; c Department of Chemistry, University of Malaya, 50603 Kuala Lumpur, Malaysia

## Abstract

In the title compound, C_18_H_15_N_3_O_2_, the dihedral angle between the ring systems is 15.1 (1)°. The amino H atom is hydrogen bonded to the exocyclic O atom of the five-membered ring, forming an *S*(6) motif.

## Related literature

For the use of the title compound as the starting reactant for the synthesis of other heterocyclic systems, see: Alsubari *et al.* (2009 ▶). For a related structure, see: Ali *et al.* (2005*a* ▶,*b* ▶).
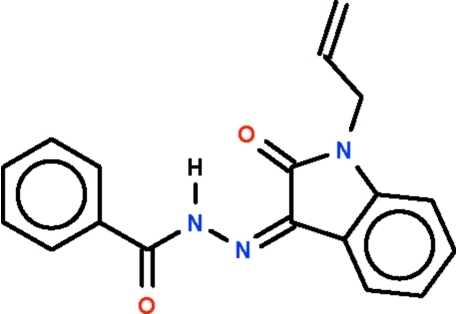


## Experimental

### 

#### Crystal data

C_18_H_15_N_3_O_2_
*M* *_r_* = 305.33
Monoclinic, 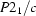

*a* = 7.5921 (2) Å
*b* = 15.1968 (4) Å
*c* = 12.8716 (3) Å
β = 94.481 (2)°
*V* = 1480.53 (7) Å^3^
*Z* = 4
Mo *K*α radiation
μ = 0.09 mm^−1^
*T* = 293 K
0.35 × 0.30 × 0.20 mm

#### Data collection

Bruker X8 APEXII diffractometer
17477 measured reflections
3286 independent reflections
2343 reflections with *I* > 2σ(*I*)
*R*_int_ = 0.059

#### Refinement

*R*[*F* ^2^ > 2σ(*F* ^2^)] = 0.041
*wR*(*F* ^2^) = 0.108
*S* = 1.00
3286 reflections
212 parameters
H atoms treated by a mixture of independent and constrained refinement
Δρ_max_ = 0.18 e Å^−3^
Δρ_min_ = −0.21 e Å^−3^

### 

Data collection: *APEX2* (Bruker, 2008 ▶); cell refinement: *SAINT* (Bruker, 2008 ▶); data reduction: *SAINT*; program(s) used to solve structure: *SHELXS97* (Sheldrick, 2008 ▶); program(s) used to refine structure: *SHELXL97* (Sheldrick, 2008 ▶); molecular graphics: *X-SEED* (Barbour, 2001 ▶); software used to prepare material for publication: *publCIF* (Westrip, 2010 ▶).

## Supplementary Material

Crystal structure: contains datablocks global, I. DOI: 10.1107/S1600536810024918/jh2174sup1.cif

Structure factors: contains datablocks I. DOI: 10.1107/S1600536810024918/jh2174Isup2.hkl

Additional supplementary materials:  crystallographic information; 3D view; checkCIF report

## Figures and Tables

**Table 1:** Hydrogen-bond geometry (Å, °)

*D*—H⋯*A*	*D*—H	H⋯*A*	*D*⋯*A*	*D*—H⋯*A*
N3—H3⋯O1	0.88 (2)	1.98 (2)	2.721 (2)	141 (2)

**Fig. 1.**
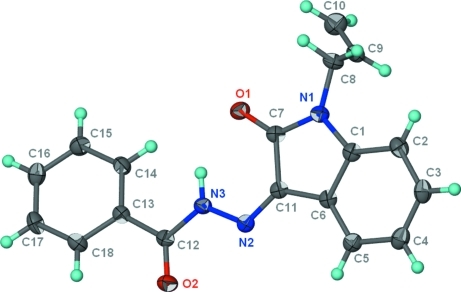
Thermal ellipsoid plot (Barbour, 2001) of the molecule of C18H15N3O2 at the 50% probability level. Hydrogen atoms are shown as spheres of arbitrary radii.

## supplementary crystallographic information

### Comment

An earlier study reports the synthesis of oxindole derivatives bearing an oxazolidin-2-one sub-unit. The title Sciff base (Scheme I) is the starting reactant for the synthesis of other heterocyclic systems (Alsubari *et al.*, 2009). We have previously determined the crystal structure of two modifications of the Schiff base derived by condensing isatin and benzoylhydrazine. In both, the amino unit of the five-membered ring functions as a hydrogen bond donor to carbonyl group of an adjacent molecule to generate a chain structure. Meanwhile, the amino –NH– unit forms an intramolecular hydrogen bond to the –C(═O)– unit of the five-membered ring (Ali *et al.*, 2005*a*, 2005*b*). The molecule of C_18_H_15_N_3_O_2_ (Fig, 1) has a phenyl ring connected to a nine-membered fused-ring through the three-atom –C(═O)–N(H)–N═unit, whose amino H-atom is hydrogen bonded to the carbonyl group of the fused-ring. The two ring systems are aligned at 15.1 (1) °.

### Experimental

1-Allylindoline-2,3-dione (0.5 g, 2.7 mmol) and benzoyl hydrazine (0.36 g, 2.7 mmol) were heated in ethanol (20 ml) for 2 h. The solvent was evaporated and the yellow solid was recrystallized from ethanol in 90% yield.

### Refinement

Carbon-bound H-atoms were placed in calculated positions (C—H 0.93–0.97 Å) and were included in the refinement in the riding model approximation, with *U*(H) set to 1.2*U*_eq_(C).

The amino H-atom was located in a difference Fourier map; the N–H distance was restrained to 0.86±0.01 Å; the temperature factor of this hydrogen atom was freely refined.

### Crystal data

**Table:**

C_18_H_15_N_3_O_2_	*F*(000) = 640
*M**_r_* = 305.33	*D*_x_ = 1.370 Mg m^−^^3^
Monoclinic, *P*2_1_/*c*	Mo *K*α radiation, λ = 0.71073 Å
Hall symbol: -P 2ybc	Cell parameters from 3004 reflections
*a* = 7.5921 (2) Å	θ = 2.7–25.0°
*b* = 15.1968 (4) Å	µ = 0.09 mm^−^^1^
*c* = 12.8716 (3) Å	*T* = 293 K
β = 94.481 (2)°	Prism, yellow
*V* = 1480.53 (7) Å^3^	0.35 × 0.30 × 0.20 mm
*Z* = 4	

### Data collection

**Table:**

Bruker X8 APEXII diffractometer	2343 reflections with *I* > 2σ(*I*)
Radiation source: fine-focus sealed tube	*R*_int_ = 0.059
graphite	θ_max_ = 27.1°, θ_min_ = 2.7°
φ and ω scans	*h* = −9→9
17477 measured reflections	*k* = −19→19
3286 independent reflections	*l* = −16→16

### Refinement

**Table:**

Refinement on *F*^2^	Primary atom site location: structure-invariant direct methods
Least-squares matrix: full	Secondary atom site location: difference Fourier map
*R*[*F*^2^ > 2σ(*F*^2^)] = 0.041	Hydrogen site location: inferred from neighbouring sites
*wR*(*F*^2^) = 0.108	H atoms treated by a mixture of independent and constrained refinement
*S* = 1.00	*w* = 1/[σ^2^(*F*_o_^2^) + (0.0463*P*)^2^ + 0.3757*P*] where *P* = (*F*_o_^2^ + 2*F*_c_^2^)/3
3286 reflections	(Δ/σ)_max_ = 0.001
212 parameters	Δρ_max_ = 0.18 e Å^−^^3^
0 restraints	Δρ_min_ = −0.21 e Å^−^^3^

### Fractional atomic coordinates and isotropic or equivalent isotropic displacement parameters (Å^2^)

**Table:**

	*x*	*y*	*z*	*U*_iso_*/*U*_eq_	
O1	0.63773 (15)	0.55475 (7)	0.60381 (8)	0.0315 (3)	
O2	0.68617 (16)	0.66838 (8)	0.24350 (8)	0.0353 (3)	
N1	0.76855 (17)	0.41763 (9)	0.62121 (10)	0.0271 (3)	
N2	0.75597 (16)	0.53877 (8)	0.38503 (9)	0.0248 (3)	
N3	0.67263 (17)	0.61477 (9)	0.40734 (10)	0.0259 (3)	
H3	0.638 (2)	0.6205 (11)	0.4710 (14)	0.034 (5)*	
C1	0.8544 (2)	0.35920 (10)	0.55565 (12)	0.0261 (3)	
C2	0.9222 (2)	0.27683 (11)	0.57850 (13)	0.0318 (4)	
H2	0.9162	0.2518	0.6441	0.038*	
C3	1.0002 (2)	0.23266 (11)	0.49925 (14)	0.0342 (4)	
H3A	1.0454	0.1765	0.5120	0.041*	
C4	1.0126 (2)	0.27008 (11)	0.40166 (14)	0.0338 (4)	
H4	1.0683	0.2395	0.3509	0.041*	
C5	0.9423 (2)	0.35293 (11)	0.37944 (12)	0.0292 (4)	
H5	0.9496	0.3781	0.3141	0.035*	
C6	0.86100 (19)	0.39725 (10)	0.45682 (12)	0.0248 (3)	
C7	0.7178 (2)	0.49230 (11)	0.56872 (12)	0.0258 (3)	
C8	0.7558 (2)	0.40781 (12)	0.73353 (12)	0.0306 (4)	
H8A	0.7490	0.3458	0.7505	0.037*	
H8B	0.6484	0.4359	0.7529	0.037*	
C9	0.9114 (2)	0.44791 (11)	0.79466 (12)	0.0302 (4)	
H9	1.0233	0.4259	0.7850	0.036*	
C10	0.8980 (3)	0.51229 (13)	0.86094 (14)	0.0418 (5)	
H10A	0.7876	0.5354	0.8720	0.050*	
H10B	0.9988	0.5350	0.8971	0.050*	
C11	0.77560 (19)	0.48267 (10)	0.46041 (11)	0.0238 (3)	
C12	0.6459 (2)	0.67879 (11)	0.33277 (11)	0.0255 (3)	
C13	0.5594 (2)	0.76046 (10)	0.36850 (11)	0.0254 (3)	
C14	0.5437 (2)	0.78064 (11)	0.47319 (12)	0.0300 (4)	
H14	0.5883	0.7420	0.5248	0.036*	
C15	0.4623 (2)	0.85771 (12)	0.50059 (13)	0.0351 (4)	
H15	0.4523	0.8708	0.5705	0.042*	
C16	0.3957 (2)	0.91536 (11)	0.42413 (14)	0.0339 (4)	
H16	0.3399	0.9669	0.4427	0.041*	
C17	0.4119 (2)	0.89656 (11)	0.32019 (13)	0.0338 (4)	
H17	0.3677	0.9356	0.2689	0.041*	
C18	0.4937 (2)	0.81987 (11)	0.29260 (13)	0.0313 (4)	
H18	0.5051	0.8077	0.2226	0.038*	

### Atomic displacement parameters (Å^2^)

**Table:**

	*U*^11^	*U*^22^	*U*^33^	*U*^12^	*U*^13^	*U*^23^
O1	0.0376 (6)	0.0279 (6)	0.0296 (6)	0.0016 (5)	0.0074 (5)	−0.0031 (5)
O2	0.0445 (7)	0.0378 (7)	0.0243 (6)	0.0041 (6)	0.0065 (5)	0.0008 (5)
N1	0.0309 (7)	0.0272 (7)	0.0234 (6)	−0.0021 (6)	0.0028 (5)	−0.0006 (5)
N2	0.0254 (7)	0.0229 (7)	0.0258 (6)	−0.0022 (5)	0.0005 (5)	−0.0037 (5)
N3	0.0325 (7)	0.0226 (7)	0.0230 (7)	0.0006 (6)	0.0036 (5)	−0.0020 (6)
C1	0.0244 (8)	0.0247 (8)	0.0286 (8)	−0.0056 (7)	−0.0011 (6)	−0.0035 (6)
C2	0.0336 (9)	0.0272 (9)	0.0338 (9)	−0.0038 (7)	−0.0015 (7)	0.0037 (7)
C3	0.0342 (9)	0.0223 (9)	0.0452 (10)	0.0013 (7)	−0.0030 (8)	−0.0014 (7)
C4	0.0323 (9)	0.0284 (9)	0.0402 (10)	0.0022 (7)	0.0003 (7)	−0.0094 (7)
C5	0.0292 (8)	0.0296 (9)	0.0283 (8)	−0.0014 (7)	0.0000 (7)	−0.0052 (7)
C6	0.0239 (7)	0.0231 (8)	0.0267 (8)	−0.0042 (6)	−0.0013 (6)	−0.0028 (6)
C7	0.0260 (8)	0.0260 (9)	0.0254 (8)	−0.0051 (7)	0.0023 (6)	−0.0018 (6)
C8	0.0366 (9)	0.0315 (9)	0.0241 (8)	−0.0049 (7)	0.0048 (7)	0.0037 (7)
C9	0.0319 (9)	0.0327 (10)	0.0259 (8)	0.0010 (7)	0.0017 (7)	0.0056 (7)
C10	0.0400 (10)	0.0462 (12)	0.0386 (10)	−0.0019 (9)	−0.0007 (8)	−0.0081 (9)
C11	0.0237 (7)	0.0225 (8)	0.0249 (8)	−0.0048 (6)	0.0007 (6)	−0.0036 (6)
C12	0.0251 (8)	0.0273 (9)	0.0237 (8)	−0.0033 (7)	0.0001 (6)	−0.0002 (6)
C13	0.0258 (8)	0.0239 (8)	0.0262 (8)	−0.0030 (6)	0.0000 (6)	0.0003 (6)
C14	0.0339 (9)	0.0300 (9)	0.0255 (8)	0.0023 (7)	−0.0005 (7)	0.0028 (7)
C15	0.0401 (10)	0.0343 (10)	0.0309 (9)	0.0022 (8)	0.0034 (7)	−0.0056 (7)
C16	0.0319 (9)	0.0248 (9)	0.0441 (10)	0.0013 (7)	−0.0018 (7)	−0.0034 (7)
C17	0.0361 (9)	0.0258 (9)	0.0380 (9)	−0.0011 (7)	−0.0066 (7)	0.0070 (7)
C18	0.0378 (9)	0.0292 (9)	0.0265 (8)	−0.0036 (7)	−0.0006 (7)	0.0021 (7)

### Geometric parameters (Å, °)

**Table:**

O1—C7	1.2318 (18)	C7—C11	1.501 (2)
O2—C12	1.2223 (18)	C8—C9	1.497 (2)
N1—C7	1.361 (2)	C8—H8A	0.9700
N1—C1	1.418 (2)	C8—H8B	0.9700
N1—C8	1.4641 (19)	C9—C10	1.307 (2)
N2—C11	1.2915 (19)	C9—H9	0.9300
N2—N3	1.3584 (18)	C10—H10A	0.9300
N3—C12	1.371 (2)	C10—H10B	0.9300
N3—H3	0.883 (18)	C12—C13	1.493 (2)
C1—C2	1.377 (2)	C13—C18	1.394 (2)
C1—C6	1.402 (2)	C13—C14	1.396 (2)
C2—C3	1.392 (2)	C14—C15	1.383 (2)
C2—H2	0.9300	C14—H14	0.9300
C3—C4	1.389 (2)	C15—C16	1.383 (2)
C3—H3A	0.9300	C15—H15	0.9300
C4—C5	1.388 (2)	C16—C17	1.383 (2)
C4—H4	0.9300	C16—H16	0.9300
C5—C6	1.387 (2)	C17—C18	1.380 (2)
C5—H5	0.9300	C17—H17	0.9300
C6—C11	1.453 (2)	C18—H18	0.9300
			
C7—N1—C1	110.61 (12)	C9—C8—H8B	109.3
C7—N1—C8	122.50 (13)	H8A—C8—H8B	108.0
C1—N1—C8	126.35 (14)	C10—C9—C8	123.28 (16)
C11—N2—N3	115.52 (13)	C10—C9—H9	118.4
N2—N3—C12	120.10 (13)	C8—C9—H9	118.4
N2—N3—H3	117.1 (11)	C9—C10—H10A	120.0
C12—N3—H3	122.8 (11)	C9—C10—H10B	120.0
C2—C1—C6	121.98 (15)	H10A—C10—H10B	120.0
C2—C1—N1	128.60 (15)	N2—C11—C6	126.25 (14)
C6—C1—N1	109.42 (14)	N2—C11—C7	127.48 (14)
C1—C2—C3	117.11 (15)	C6—C11—C7	106.27 (13)
C1—C2—H2	121.4	O2—C12—N3	122.15 (15)
C3—C2—H2	121.4	O2—C12—C13	123.06 (14)
C4—C3—C2	121.89 (16)	N3—C12—C13	114.77 (13)
C4—C3—H3A	119.1	C18—C13—C14	118.77 (15)
C2—C3—H3A	119.1	C18—C13—C12	117.67 (14)
C5—C4—C3	120.37 (16)	C14—C13—C12	123.55 (14)
C5—C4—H4	119.8	C15—C14—C13	120.38 (15)
C3—C4—H4	119.8	C15—C14—H14	119.8
C6—C5—C4	118.56 (15)	C13—C14—H14	119.8
C6—C5—H5	120.7	C14—C15—C16	120.05 (16)
C4—C5—H5	120.7	C14—C15—H15	120.0
C5—C6—C1	120.06 (15)	C16—C15—H15	120.0
C5—C6—C11	132.96 (15)	C17—C16—C15	120.14 (16)
C1—C6—C11	106.97 (13)	C17—C16—H16	119.9
O1—C7—N1	126.09 (14)	C15—C16—H16	119.9
O1—C7—C11	127.17 (14)	C18—C17—C16	119.95 (16)
N1—C7—C11	106.74 (13)	C18—C17—H17	120.0
N1—C8—C9	111.54 (13)	C16—C17—H17	120.0
N1—C8—H8A	109.3	C17—C18—C13	120.69 (15)
C9—C8—H8A	109.3	C17—C18—H18	119.7
N1—C8—H8B	109.3	C13—C18—H18	119.7
			
C11—N2—N3—C12	179.73 (14)	N3—N2—C11—C7	−0.3 (2)
C7—N1—C1—C2	−178.87 (15)	C5—C6—C11—N2	0.6 (3)
C8—N1—C1—C2	9.5 (3)	C1—C6—C11—N2	179.33 (14)
C7—N1—C1—C6	0.38 (17)	C5—C6—C11—C7	−178.78 (16)
C8—N1—C1—C6	−171.30 (14)	C1—C6—C11—C7	−0.03 (16)
C6—C1—C2—C3	0.7 (2)	O1—C7—C11—N2	1.9 (3)
N1—C1—C2—C3	179.84 (15)	N1—C7—C11—N2	−179.10 (14)
C1—C2—C3—C4	1.1 (2)	O1—C7—C11—C6	−178.79 (15)
C2—C3—C4—C5	−1.7 (3)	N1—C7—C11—C6	0.26 (16)
C3—C4—C5—C6	0.4 (2)	N2—N3—C12—O2	4.0 (2)
C4—C5—C6—C1	1.4 (2)	N2—N3—C12—C13	−177.63 (12)
C4—C5—C6—C11	179.98 (16)	O2—C12—C13—C18	11.9 (2)
C2—C1—C6—C5	−1.9 (2)	N3—C12—C13—C18	−166.38 (14)
N1—C1—C6—C5	178.75 (13)	O2—C12—C13—C14	−167.09 (15)
C2—C1—C6—C11	179.12 (14)	N3—C12—C13—C14	14.6 (2)
N1—C1—C6—C11	−0.19 (16)	C18—C13—C14—C15	0.8 (2)
C1—N1—C7—O1	178.68 (15)	C12—C13—C14—C15	179.84 (15)
C8—N1—C7—O1	−9.3 (2)	C13—C14—C15—C16	0.0 (3)
C1—N1—C7—C11	−0.38 (16)	C14—C15—C16—C17	−0.6 (3)
C8—N1—C7—C11	171.67 (13)	C15—C16—C17—C18	0.4 (3)
C7—N1—C8—C9	−83.08 (19)	C16—C17—C18—C13	0.5 (3)
C1—N1—C8—C9	87.67 (19)	C14—C13—C18—C17	−1.1 (2)
N1—C8—C9—C10	119.36 (18)	C12—C13—C18—C17	179.85 (14)
N3—N2—C11—C6	−179.51 (13)		

### Hydrogen-bond geometry (Å, °)

**Table:**

*D*—H···*A*	*D*—H	H···*A*	*D*···*A*	*D*—H···*A*
N3—H3···O1	0.88 (2)	1.98 (2)	2.721 (2)	141 (2)
